# An Accurate and Precise ddPCR-Based Method for Determining the Concentration of Plasmid DNA

**DOI:** 10.21769/BioProtoc.5741

**Published:** 2026-07-20

**Authors:** Franco Puleo, Annicka Evans, Cullen Mason

**Affiliations:** Technical Development, Biogen, Cambridge, MA, USA

**Keywords:** Plasmid DNA, Droplet digital PCR, DNA linearization, rAAV manufacturing, Gene therapy workflows, Selection cassette targeting, Process development

## Abstract

Transient transfection is commonly used for the commercial production of adeno-associated viral particles for gene therapy. In this process, packaging cells such as HEK293 cells are transfected with three plasmids, including the Rep/Cap plasmid, the Helper plasmid, and the gene-of-interest plasmid containing the transgene/gene therapy product. The combination of these plasmids allows for the robust production of recombinant adeno-associated viral particles. As a result, the concentration of these plasmids plays a critical role in viral production and must be accurately assessed. Typically, A_260_/A_280_ readings are utilized to measure plasmid titer; however, this approach lacks accuracy and specificity and is susceptible to matrix interference. To address these shortcomings, a digital droplet PCR method was developed to titer plasmids. This method uses a combined restriction digest/PCR protocol to linearize the plasmid template and evaluate copy numbers of a plasmid-specific gene. Qualification demonstrated that the method is highly accurate, specific to plasmid DNA, and impervious to matrix interference.

Key features

• Digital droplet PCR (ddPCR)-based plasmid quantification using a combined linearization/PCR workflow, enabling highly accurate and precise copy-number measurements across plasmids of varying purity and production stages.

• Robust performance in complex sample matrices, with high dilution tolerance that minimizes matrix interference and ensures reliable quantification from crude lysates to highly purified plasmids.

• Specific detection of plasmid DNA independent of contaminating nucleic acids, providing reliable readouts when A_260_ measurements are distorted by residual DNA, RNA, or dNTP impurities.

• Universally applicable to plasmids sharing common selection cassettes, enabling a single primer–probe set to accurately quantify diverse constructs without plasmid-specific assay redesign.

## Graphical overview


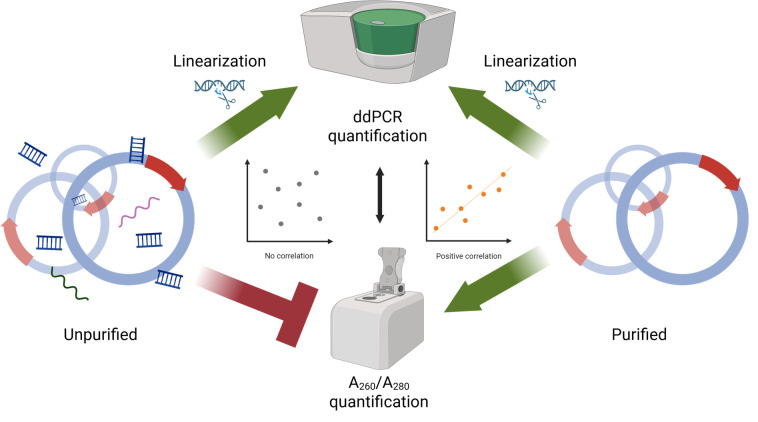


**Digital droplet PCR (ddPCR) enables accurate, matrix-independent plasmid quantification for adeno-associated virus (AAV) production, outperforming traditional A_260_/A_280_ measurements**

## Background

Recombinant adeno-associated virus (rAAV) is a popular avenue for the development of gene therapy products, given its low safety risk and long-lasting transgene expression [1]. Production of rAAV particles can occur by multiple means; however, transient transfection (TT) remains at the forefront. The TT process utilizes three plasmids containing the necessary elements for rAAV production: replication and capsid genes (rep/cap), helper genes that support packaging without the need for helper virus, and the packaged transgene needed for therapeutic effect [1]. Adherent or suspension cells are simultaneously transfected with the three plasmids and ultimately generate rAAV that serves as the gene therapy product [1,2]. The ratio of TT plasmids [rep/cap, helper, and gene of interest (GOI)] significantly influences rAAV yield and the quality of the rAAV product; therefore, accurate plasmid concentration is a critical component of the TT process [2]. Plasmid DNA is frequently measured by taking the absorbance at 260 nm (A_260_) in a similar fashion to native DNA, as it is a quick approach that requires a spectrophotometer and as little as a few microliters of sample. Although the A_260_ readings provide insight into plasmid concentration, these titers are not reliable, as the readout is a measure of total nucleic acid that is not specific to plasmid DNA. The presence of residual host cell DNA or RNA can artificially inflate plasmid titer. Moreover, the sample matrix itself can also significantly impact the quality of the data, effects that are most severe in impure samples. These limitations of the A_260_ approach highlight the need for a more accurate and precise approach for evaluating plasmid concentration directly.

To address this need, we developed a simplified, one-step digital droplet PCR (ddPCR) assay that couples plasmid linearization with amplification and quantifies plasmid DNA using primers/probe targeting the antibiotic resistance cassette. Compared to quantitative PCR (qPCR), ddPCR enables absolute quantification without reliance on standard curves and is less sensitive to amplification efficiency and inhibitors. This approach is user-friendly as it allows for a single set of primers and probe to be used with multiple plasmid types. Given that the method is specific to plasmids, titers are not susceptible to inflation due to residual host cell DNA/RNA. Furthermore, the sensitivity of ddPCR requires substantial sample dilution to obtain copy numbers within the analytical range and results in negligible matrix interference regardless of sample purity. Plasmid preparations were tested using ddPCR and demonstrated that the method is extremely accurate and precise. Collectively, our findings suggest that our ddPCR method is suitable for evaluating plasmid concentration not only in the context of plasmid purification or rAAV generation by TT but also in any scenario requiring highly accurate and precise readouts.

## Materials and reagents

**Biological materials**

1. Plasmids rep/cap, helper, and GOI, produced internally by *E. coli*, followed by alkaline lysis and column-based purification (e.g., Qiagen HiSpeed Plasmid Kit, catalog number: 12643)

*Note: Most commercial or in-house plasmids will work with this method if a sequence-specific primer is designed.*

**Reagents**

1. 2× ddPCR Supermix for probes (no dUTP) (Bio-Rad, catalog number: 186 3025)

2. Automated droplet generation oil for probes (Bio-Rad, catalog number: 186 4110)

3. Droplet reader oil (Bio-Rad, catalog number: 186 3004)

4. NdeI restriction enzyme (New England BioLabs, catalog number: R0111L)

5. Primer/probes (Thermo Fisher Scientific, custom); kanamycin resistance (KANR) primer/probe:

a. Forward primer: GGG CTT CCC ATA CAA TCG ATA G

b. Reverse primer: GGT ATA AAT GGG CTC GCG ATA A

c. Probe: TTG TCG CAC CTG ATT GCC CGA C

*Note: The KANR primer probe was designed to target the KANR cassette commonly used for selection*.

6. Reagent reservoirs (undivided and divided) (Vistalab, catalog numbers: 3054 1002, 3054 1004)

7. TE buffer, pH 8.0 (Thermo Fisher Scientific, catalog number: AM9849)

8. Ultrapure distilled water (DNase/RNase free) (Thermo Fisher Scientific, catalog number: 10977)

**Solutions**

1. Primers probe set specific to target cassette (see Recipes)

2. Combined linearization/ddPCR reaction master mix (see Recipes)

**Recipes**

**1. Primers probe set specific to target cassette**

**Table:** 

Reagent	Stock concentration (μM)	Volume stock (μL)	Final concentration (μM) in TE
Forward primer	90	300	30
Reverse primer	90	300	30
Probe	25	300	8.33

The lyophilized primers are reconstituted to 90 μM in TE, and the probes are received at 100 μM. The probe is diluted down to 25 μM in TE. The primers and probe are then combined in equal volumes. An example is provided above. Store at -20 °C.

**2. Combined linearization/ddPCR reaction master mix**

**Table:** 

Reagent	Stock concentration	Reaction volume (μL)	Final concentration
ddPCR Supermix for probes	2×	12.5	1×
Primers probe set (Recipe 1)	30 μM forward/reverse primers, 8.33 μM probe	0.75	0.9 μM forward/reverse primers, 0.010 μM probe
Water		1.55	
Restriction enzyme	20,000 units/mL	0.2	160 units/mL
Sample		10	
Total		25	

Prepare fresh and store on ice until use.

**Laboratory supplies**

1. 1.5 mL DNA LoBind microcentrifuge tubes (Eppendorf, catalog number: 925000064)

2. 15 mL sterile polypropylene conical tube (Corning or equivalent, catalog number: 430052)

3. 96-well, not treated or tissue culture treated, round-bottom microplates (Corning, catalog number: 3359 or 3799)

4. ddPCR 96-well PCR plates (Bio-Rad, catalog number: 12001925)

5. DG32 automated droplet generator cartridges (Bio-Rad, catalog number: 186 4109)

6. Pierceable foil heat seal (Bio-Rad, catalog number: 181 4040)

7. Pipette tips for the AutoDG system (Bio-Rad, catalog number: 186 4120)

8. Pipette tips, sterile filter (any)

## Equipment

1. C1000 Touch^TM^ Thermal Cycler with 96-Deep Well Reaction Module (Bio-Rad, catalog number: 1851197)

2. Mini Centrifuge (Labnet, catalog number: C1301; or equivalent)

3. Mini Plate Spinner (Fisher Scientific, catalog number: 14100143; or equivalent)

4. PX1^TM^ PCR Plate Sealer (Bio-Rad, catalog number: 1814000)

5. QX200^TM^ Automated Droplet Generator (Bio-Rad, catalog number: 1864101)

6. QX200^TM^ Digital PCR Reader (Bio-Rad, catalog number: 1864003)

7. Vortex mixer (Fisher Scientific, catalog number: 02215365; or equivalent)

## Software and datasets

1. QuantaSoft^TM^ (Bio-Rad, catalog number: 1.7.4); free to download

*Note: This was used to analyze ddPCR data.*

2. SnapGene (Dotmatics); basic version is free to download

*Note: This was used to design the KANR primers probe set.*

## Procedure

**A. Sample preparation**

1. Thaw plasmid samples, controls, 2× ddPCR supermix for probes (no dUTP), and primers probe set (Recipe 1) at room temperature. The control can be any representative plasmid of known concentration that has a cut site for the restriction enzyme that is outside of the target amplicon.

2. Place the restriction enzyme on ice immediately after removal from storage.

**Critical:** Keep the restriction enzyme on ice during reaction setup to preserve activity.

3. Vortex samples and reagents for 5–10 s to ensure a homogenous solution. Briefly spin down using a mini centrifuge.

4. Dilute plasmid samples and controls in TE in either a 96-well plate or 1.5 mL DNA LoBind microcentrifuge tubes to the desired copies/μL, ensuring that results fall within the analytical range of ddPCR (5–5,000 copies/μL).

**Example:** A plasmid with a concentration of 1 × 10^10^ copies/mL should be diluted 1000-fold in TE (10 μL of plasmid in 990 μL of TE) to 1 × 10^7^ copies/mL. The subsequent addition of the ddPCR master mix (see step A7) will result in a 2.5-fold dilution and a final concentration of 4 × 10^6^ copies/mL, which is equivalent to 4,000 copies/μL.

*Note: Prepare at least 50 μL of each dilution to accommodate replicate wells and pipetting dead volume.*

5. Prepare ddPCR master mix according to Table 1; vortex for 5–10 s to ensure a homogenous solution.

*Notes:*

*1. ddPCR master mix can be scaled to accommodate multiple samples and prepared in a 15 mL conical tube if needed.*

*2. Clean droplet separation with minimal rain confirmed that NdeI is compatible with the ddPCR master mix; therefore, the digestion can take place within the ddPCR reaction. Restriction enzyme compatibility should be confirmed if other restriction enzymes are used.*

**Table 1.** Digital droplet PCR (ddPCR) master mix preparation

Reagent	Volume per reaction (μL)
2× ddPCR Supermix for probes	12.5
Primers probe set (30 μM forward/reverse primers, 8.33 μM probe)	0.75
Water	1.55
Restriction enzyme (20,000 units/mL)	0.2
Total volume per reaction	15

6. Add 10 μL of sample or control to a well of a 96-well ddPCR plate.

*Note: The expected copy number should be within the ddPCR analytical range: 5–5,000 copies/μL.*

7. Add 15 μL of ddPCR master mix to each well containing the sample to make a final ddPCR reaction volume of 25 μL.

**B. ddPCR**

1. Seal the plate with foil using the PX1^TM^ PCR plate sealer.

2. Vortex the sealed plate for 10 s and briefly spin down at 2,500 rpm using a mini plate spinner to ensure the reaction is at the bottom of the well.

3. Load the ddPCR plate, droplet generator cartridge, pipette tips, reagent reservoirs, and automated droplet generation oil for probes into the QX200^TM^ Automated Droplet Generator. Then, start the droplet generation run using the instrument’s standard droplet generation program.

**Critical:** Confirm that each well generates droplets and that no wells are flagged by the instrument before proceeding.

4. Upon completion of droplet generation, seal the droplet plate with a new foil using PX1^TM^ PCR plate sealer.

5. Place the sealed plate in a C1000 Touch^TM^ thermal cycler (96-deep well reaction module) and run the PCR program shown in Table 2.

**Pause point:** After the PCR program finishes, you may hold the plate at 4 °C in the thermocycler (Table 2, “Hold” step) until you are ready to read the plate.

**Table 2.** PCR protocol

Step	Temperature (°C)	Time	Number of cycles
Linearization	37	15 min	1
Enzyme activation	95	10 min	1
Denature	94	30	40
Annealing	60	1 min	
Enzyme deactivation	98	10 min	1
Hold	4	∞	1

6. Upon completion of PCR, place the sample plate in the QX200^TM^ Digital PCR Reader.

7. Open QuantSoft^TM^ software or equivalent. Set up a new plate according to the plate layout and run according to the manufacturer’s instructions.

8. Analyze each well in QuantaSoft^TM^ by applying either (a) a manual fluorescence threshold or (b) the software’s automated thresholding function. Then, export the calculated concentration (copies/μL) for each sample. If you observe extensive droplet rain or unclear separation between positive and negative droplets, see General notes and troubleshooting.

9. Call droplets above the negative-droplet fluorescence band as positive droplets for the target and then review replicate wells to confirm consistent separation and concentration.

10. Export raw data as a CSV and continue analysis in a spreadsheet software such as Excel.

## Data analysis

Plasmid titer for a sample is calculated using the following equation:

Plasmid titer copiesmL=measured KANR copiesμL×Dilution Factor×2.5 ddPCR dilution×1000μLmL

Theoretical copy numbers were estimated for the linearity assessment using the following equation:

Theoretical KANRcopiesμL=plasmid titer KANRcopiesμLdilution factor

In all cases, recovery was calculated by the following equation:

$$Recovery\;\%=\frac{Measured}{Theoretical}\times 100\%$$

The ratio of ddPCR concentrations to A_260_ concentrations expressed as % A_260_ was calculated using the following equation:

% A260=ddPCR concentrationngμLA260 concentrationngμL×100%

The theoretical ddPCR concentration, based on applying a correction factor to the A_260_ concentration, was calculated using the following equation:

Theoretical ddPCR concentrationngμL=A260ngμL×correction factor

The recovered spike value for a particular sample dilution was calculated by the following equation:

Recovered Spike KANR copiesμL=Spiked KANRcopiesμL-Unspiked KANRcopiesμL

Spike recovery for a particular sample dilution was calculated using the following equation:

Spike recovery %= recovered Spike KANR copiesμLMeasured Spike KANR copiesμL

The relative standard deviation (RSD) was calculated by the following formula:

$$RSD\;\%=100\%\times \frac{standard\;deviation\;(SD)}{Average}$$

**Development of a single-step protocol**

**Effect of the DNA tertiary structure**

**Figure 1.**
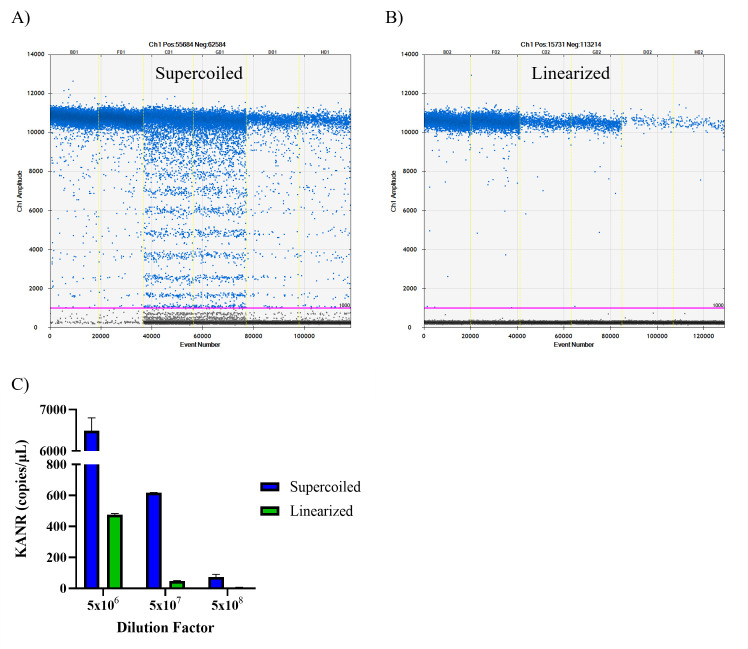
Digital droplet PCR (ddPCR) of supercoiled vs. linearized plasmid. Intact supercoiled rep/cap plasmid and rep/cap plasmid linearized using NdeI restriction enzyme were evaluated using ddPCR targeting the KANR cassette. (A) ddPCR droplet plots for supercoiled and (B) linearized rep/cap plasmid. (C) Quantification of KANR copy numbers (copies/μL) at various dilution factors. Supercoiled copy numbers are shown in green, and linearized copy numbers are in blue. Error bars represent standard deviation (SD), n=2.

ddPCR is a reliable method for the quantification of DNA copy numbers. However, the conformation of plasmid DNA affects ddPCR efficiency, as it is well known that hybridization of primers and probe to a supercoiled plasmid DNA is compromised [3]. To confirm the need for linearization of the plasmid template, rep/cap plasmid (pRep/Cap) was treated with or without NdeI restriction enzyme (single cutter) and subsequently serially diluted 10-fold, starting at a dilution factor of 5 × 10^6^ and ending at 5 × 10^8^ prior to ddPCR. Supercoiled plasmid produced substantial droplet rain and poor separation between positive and negative populations (Figure 1A), making thresholding difficult and reducing confidence in the data. In contrast, linearization resolved droplet rain and yielded clean, well-separated droplet populations (Figure 1B). The supercoiled droplet pattern can also serve as a useful indicator of incomplete or failed linearization (see Troubleshooting section). Consistent with these observations, KANR copy numbers—plotted according to the dilution factor used—were markedly higher in supercoiled plasmid compared to linearized plasmid (Figure 1C), likely reflecting errors introduced during thresholding under conditions of poor droplet resolution. These results demonstrate that plasmid linearization is essential for accurate ddPCR quantification. To address this, we developed a user-friendly, one-step approach that incorporates restriction enzyme-mediated linearization directly into the ddPCR workflow, requiring only the addition of the enzyme to the reaction mix and a modest extension of thermocycling time.

## Validation of protocol

**A. Accuracy and precision**

Plasmids are produced by transformation into competent bacterial cells such as *E. coli* and amplification in the presence of antibiotics, followed by alkaline lysis and column-based purification. To assess the accuracy, precision, and matrix robustness of the ddPCR method for informing plasmid production, samples were taken at varying points in the purification process. Samples comprised of crude lysates, clarified lysates, the pH neutralization step, and the final purified GOI plasmid were tested across a range of dilutions in the presence or absence of a pRep/Cap spike (Table 3).

All samples demonstrated acceptable spike recovery (80%–120% of the spike control: 449 copies/μL) in at least three sample dilutions, confirming assay accuracy across purification stages. The overall assay accuracy, calculated as the average of all acceptable spike recoveries, was 104%. Precision was evaluated both at the sample level and across the assay: plasmid titer precision for individual samples, expressed as %RSD, was less than 4%, while overall assay precision, calculated as the RSD of all acceptable spike recoveries, was 5%.

To further evaluate matrix effects, plasmid samples were assessed across dilutions. While less purified samples showed poor spike recovery at the lowest dilutions (Table 3, crude lysate), this effect was mitigated at higher dilutions, where matrix interference was minimized, and accurate titers were obtained. Importantly, these crude lysate samples represent unpurified plasmid preparations, and acceptable recovery observed at higher dilutions supports that the ddPCR method performs reliably in unpurified sample matrices. Given the high sensitivity of ddPCR, substantial dilution can be supported, minimizing matrix interference as long as concentrations remain above 5 copies/μL. Collectively, these results demonstrate that the method is accurate, precise, and robust, and can be broadly applied to plasmid samples generated under diverse conditions without requiring extensive purification.

**Table 3.** Accuracy and precision of the digital droplet PCR (ddPCR) plasmid titer method. Four plasmid samples increasing in purity were tested using multiple dilutions in ddPCR. In this experiment, process step samples were treated with and without a rep/cap plasmid spike. Recovered spike copy number (KANR copies/μL) was calculated as the copy number of the spiked sample subtracted by the copy number of the unspiked sample for a particular dilution. Spike recovery was determined as the spike copy number divided by the measured spike value and expressed as a percentage. Plasmid concentration for each dilution was calculated by multiplying the dilution factor, the ddPCR dilution factor (2.5), and the unspiked copy number and converting to copies/mL. The precision for each plasmid titer was calculated as the RSD of the plasmid concentrations with acceptable spike recoveries (80%–120% of the measured spike value). Assay accuracy was calculated as the average of all acceptable spike recoveries and expressed as a percentage. Assay precision was calculated as the RSD of all acceptable spike recoveries.

Process step	Dilution factor	Unspiked KANR (copies/μL)	Spiked KANR (copies/μL)	Spike recovery (%)	Plasmid titer KANR (copies/mL)	RSD (%)
Crude lysate	4.17×10^5^	4,020	105	23	1.68×10^12^	2.7
	1.67×10^6^	953	488	108	1.59×10^12^	
	6.67×10^6^	244	462	103	1.63×10^12^	
	2.67×10^7^	58	481	107	1.54×10^12^	
Clarified lysate	3.29×10^5^	4,390	715	159	1.44×10^12^	2.0
	1.32×10^6^	1,063	500	111	1.40×10^12^	
	5.26×10^6^	266	468	104	1.40×10^12^	
	2.11×10^7^	69	471	105	1.45×10^12^	
pH neutralization	1.56×10^5^	4,270	430	96	6.67×10^11^	3.5
	6.25×10^5^	1,058	410	91	6.61×10^11^	
	2.50×10^6^	259	460	102	6.46×10^11^	
	1.00×10^7^	62	471	105	6.16×10^11^	
Purified plasmid	2.50×10^7^	5,035	645	143	1.26×10^14^	0.10
	1.00×10^8^	1,186	497	111	1.19×10^14^	
	4.00×10^8^	296	478	106	1.18×10^14^	
	1.60×10^9^	74	481	107	1.19×10^14^	
pRep/Cap spike	-	-	449	-	-	-
		Accuracy		104%		
		Precision		5%		

**B. Assay linearity**

**Figure 2.**
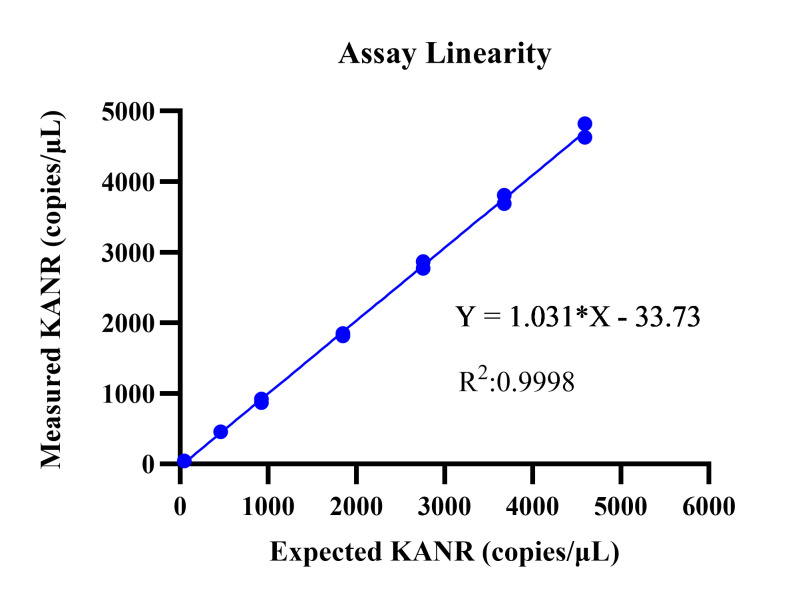
Linearity of the digital droplet PCR (ddPCR) plasmid titer method. The rep/cap plasmid was diluted to obtain KANR copy numbers within the range of ddPCR (5–5,000 copies/μL). Measured copy numbers were plotted against expected copy numbers using a linear fit.

The linear range of the ddPCR method was determined by diluting the rep/cap plasmid within the analytical range of ddPCR (5 copies/μL to 5000 copies/μL). Assay linearity was demonstrated by graphing the theoretical copies/μL vs. the experimentally determined copies/μL (Figure 2). Only dilutions that had acceptable recovery (70%–130% of the theoretical value) were used in calculating linearity (Table 4). The assay is linear throughout the ddPCR analytical range. The slope of the linear regression was 1.031, and the coefficient of determination was >0.999.

**Table 4.** Measured and expected KANR copy numbers. The rep/cap plasmid was diluted to obtain KANR copy numbers within the range of digital droplet PCR (ddPCR) (5–5000 copies/μL). Expected copy numbers were based on dilution of the measured rep/cap plasmid concentration. Recovery was calculated as expected copy numbers divided by measured copies and expressed as a percentage.

Dilution #	Expected (copies/μL)	Measured (copies/μL)	Recovery
1	4,595	4,630	101%
2	3,676	3,750	102%
3	2,757	2,820	102%
4	1,847	1,833	99%
5	924	896	97%
6	462	453	98%
7	46	45	97%
8	5	7	143%

**C. Specificity**

Assay specificity was evaluated using the purified plasmid in combination with common plasmid impurities. In this experiment, the purified plasmid was spiked with increasing amounts of *E. coli* genomic DNA, *E. coli* RNA, and dNTPs. Sample concentrations were determined by measuring the absorbance at A_260_ and by ddPCR. Copy numbers obtained by ddPCR were converted to ng/μL using the following equation:

copiesmL×650 gmolbasepairs×#bp×1×109ngg6.022×1023copiesmol×1000 μLmL

The average molar mass of DNA is 650 (g/mol) per base pair (bp). Using the length of the plasmid in base pairs and Avogadro’s number, copies/mL can be converted to ng/μL. With this equation, ddPCR results can be compared to other quantitative methods for determining DNA concentrations.

**Figure 3.**
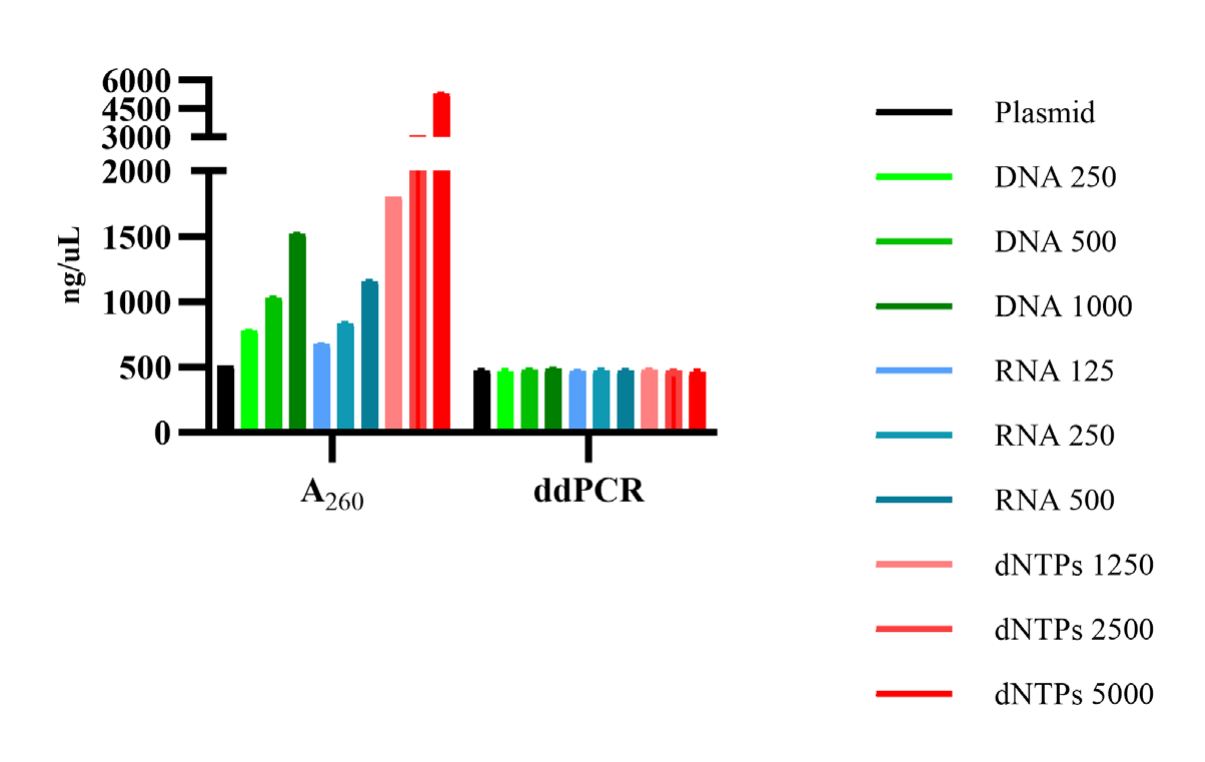
Specificity of the digital droplet PCR (ddPCR) plasmid titer method. Rep/Cap plasmid samples diluted to a single concentration were spiked with *E. coli* DNA, *E. coli* RNA, or dNTPs. Concentration was measured by A_260_ and ddPCR. Error bars represent SD, n=3.

The A_260_ measurements were significantly impacted by the presence of all spikes, with apparent concentrations increasing proportionally to the amount of impurity added (Figure 3, Table 5). In contrast, ddPCR-derived plasmid titers remained unchanged across all conditions, demonstrating that residual impurities did not affect the assay readout. These results highlight the lack of specificity of spectrophotometric methods such as NanoDrop, which detect total nucleic acid content, and reinforce that ddPCR provides specific and accurate quantification of plasmid DNA even in the presence of impurities. This specificity is driven by the use of primers and probes targeting plasmid-specific sequences. While this requires the generation of plasmid-specific primer sets, the approach can be generalized by targeting common elements.

In this study, the KANR cassette was selected as a universal target, enabling quantification of multiple TT plasmid types without the need for plasmid-specific primers. The inclusion of rep/cap, helper, and GOI plasmids demonstrates applicability across multiple plasmid types sharing the KANR cassette. A limitation of this approach is the inability to distinguish between mixtures of plasmids sharing the same target sequence; however, advances in ddPCR multiplexing provide a path forward for simultaneous detection using multiple primer/probe sets. Additionally, by targeting unique plasmid sequences, this method can be adapted for plasmid identity confirmation during quality assessment of rAAV TT starting materials.

**Table 5.** Assay specificity. *E. coli* DNA and RNA along with dNTPs were spiked into purified plasmid samples and measured by A_260_ and digital droplet PCR (ddPCR) plasmid titer (copy numbers were converted to ng/μL for comparison).

Spike material	Spike concentration (ng/μL)	Avg measured A_260_ (ng/μL)	Avg ddPCR (ng/μL)
Plasmid only		509	474
DNA	250	782	466
	500	1,034	479
	1,000	1,520	487
RNA	125	674	468
	250	838	473
	500	1,159	473
dNTPs	1,250	1,803	478
	2,500	3,113	475
	5,000	5,312	462

**D. A_260_ method comparison in purified plasmids**

**Figure 4.**
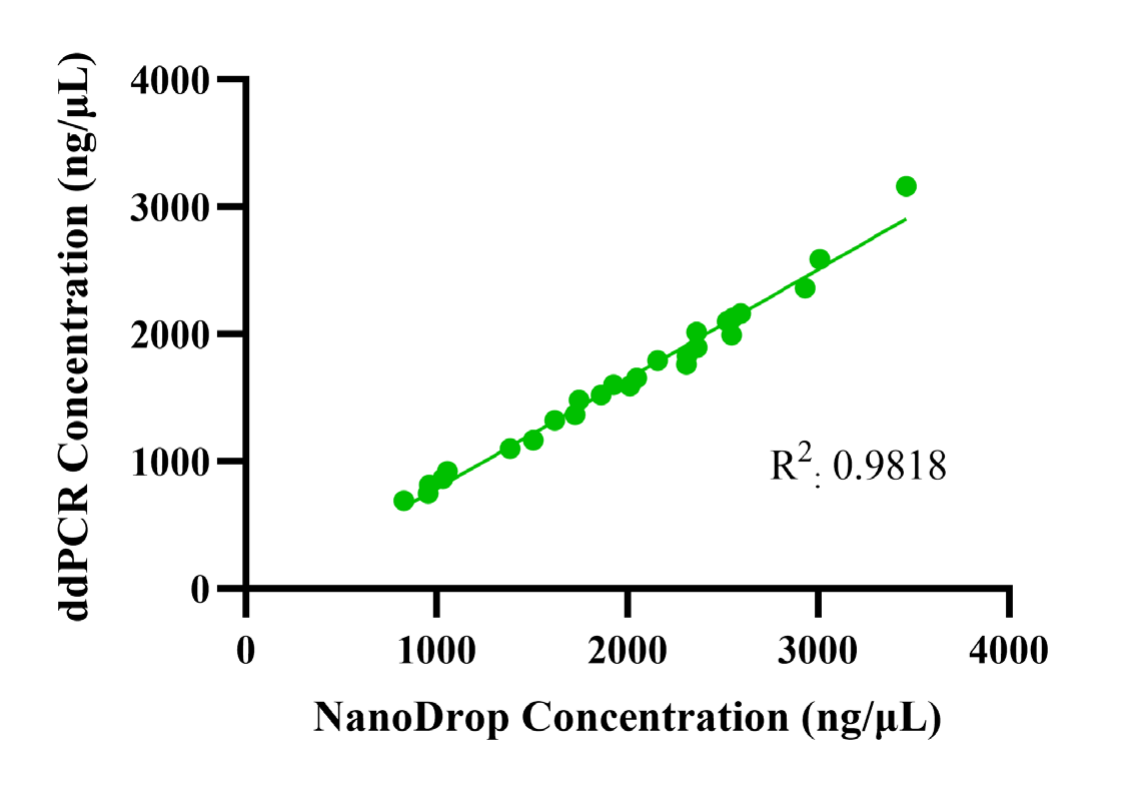
Correlation between digital droplet PCR (ddPCR) concentrations and A_260_ concentrations for purified plasmids. Plasmid concentrations determined via ddPCR were plotted against concentrations determined via the A_260_ method using a linear fit. Coefficient of determination: >0.98.

Multiple samples of purified rep/cap, helper, and GOI plasmids containing the KANR cassette were quantified by A_260_ absorbance readings and the ddPCR plasmid titer method. The A_260_-determined concentrations were consistently higher than the converted ddPCR-derived concentrations for all 26 samples (Table 6). Expressing the ratio of ddPCR- to A_260_-determined concentrations as a percentage (% A_260_) demonstrated that, on average, the ddPCR-derived concentration was 82% of the concentration by A_260_. Plotting A_260_ vs. ddPCR concentrations for the purified plasmid showed that there is a strong correlation between the two methods, with a coefficient of determination of >0.98 (Figure 4). With this equation, ddPCR results can be compared to other quantitative methods for determining DNA concentrations.

**Table 6.** A_260_ vs. digital droplet PCR (ddPCR) plasmid titer in purified plasmids. Quantification of purified plasmid by NanoDrop spectrophotometer and ddPCR plasmid titer. % A_260_ was calculated as the measured ddPCR concentration divided by the A_260_ concentration and expressed as a percentage.

Sample	ddPCR (ng/μL)	A_260_ (ng/μL)	% A_260_
1	814	962	85%
2	921	1,056	87%
3	691	827	84%
4	1761	2,310	76%
5	864	1,033	84%
6	747	956	78%
7	1,993	2,547	78%
8	1,100	1,386	79%
9	1,594	2,013	79%
10	1,366	1,726	79%
11	1,655	2,049	81%
12	1,827	2,314	79%
13	1,602	1,927	83%
14	1,522	1,862	82%
15	1,320	1,618	82%
16	2,101	2,523	83%
17	1,165	1,508	77%
18	2,362	2,932	81%
19	1,895	2,367	80%
20	1,484	1,748	85%
21	3,163	3,461	91%
22	2,589	3,009	86%
23	2,160	2,593	83%
24	1,794	2,158	83%
25	2,125	2,552	83%
26	2,016	2,363	85%
		Average % A_260_	82%
		RSD	4%

The A_260_ method for quantifying plasmid concentration is a much simpler method that can be used to quickly determine plasmid concentration. Given the strong correlation between the A_260_-determined and ddPCR-derived concentrations, it was then determined whether a correction factor could be applied to the A_260_ concentrations to accurately predict the ddPCR plasmid titer concentrations. The average ddPCR-to-A_260_ ratio (% A_260_) was used as a correction factor, and the A_260_ concentrations for a second sample set were multiplied by this factor to obtain a theoretical ddPCR-derived concentration for each of the samples (Table 7). The actual ddPCR-derived concentrations were also determined. To evaluate the accuracy of this conversion, the % recovery was calculated. The accuracy of the correction factor was calculated by averaging the % recovery of all samples and was determined to be 104%. The precision of the correction factor was calculated as the RSD of the % recovery and was determined to be 7%. Thus, a strong correlation exists between the ddPCR and absorbance-based methods for purified plasmids.

**Table 7.** A_260_ vs. digital droplet PCR (ddPCR) plasmid titer in an additional set of purified plasmids. Quantification of purified plasmid by NanoDrop spectrophotometer and ddPCR plasmid titer. % A_260_ was calculated as the measured ddPCR concentration divided by the A_260_ concentration and expressed as a percentage. Theoretical titer was calculated by multiplying the A_260_ concentration by 0.82 (correction factor). % recovery was calculated as the actual ddPCR-derived titer divided by the theoretical titer and expressed as a percentage. Note: Sample 4 was excluded for being an outlier and was not considered in the calculations.

**Sample**	**ddPCR** **(ng/μL)**	**A_260_** **(ng/μL)**	**%A_260_**	**Theoretical** **(ng/μL)**	% **recovery**
1	873	1,058	83%	868	101
2	864	1,013	85%	831	104
3	884	1,043	85%	855	103
4	549	1,065	52%	873	63
5	771	908	85%	745	104
6	855	1,022	84%	838	102
7	765	958	80%	786	97
8	805	1,014	79%	831	97
9	830	963	86%	790	105
10	788	1,062	74%	871	90
11	799	999	80%	819	97
12	881	1,057	83%	867	102
13	886	1,049	84%	860	103
14	880	1,071	82%	878	100
15	962	1,093	88%	896	107
16	803	994	81%	815	99
17	2194	2,246	98%	1,841	119
18	2986	3,233	92%	2,651	113
19	1883	1,961	96%	1,608	117
20	1921	2,075	93%	1,702	113
		Average % A_260_	85%	Accuracy	104%
		RSD	7%	Precision	7%

**E. A_260_ method comparison in unpurified plasmids**

**Figure 5.**
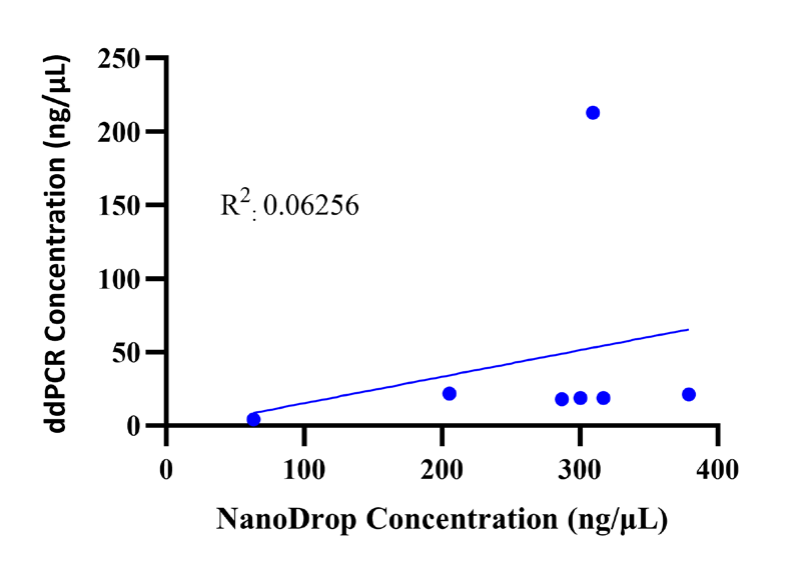
Correlation between digital droplet PCR (ddPCR) and A_260_ in unpurified plasmids. Plasmid concentrations determined via ddPCR were plotted against concentrations determined via the A_260_ using a linear fit. Coefficient of determination: <0.10.

An additional comparison study was performed using plasmid preparations varying in purity. These samples were tested using the A_260_ and ddPCR methods. The ddPCR concentrations were substantially lower than the A_260_ concentrations for these plasmids, and thus, % A_260_ ratios were less than 10% for most samples (Table 8). Comparison of the two methods showed there was no linear correlation between A_260_ and ddPCR methods for unpurified plasmids (coefficient of determination <0.10, Figure 5). The two methods aligned better with more purified material (such as sample 7), supporting the strong correlation between methods when using purified plasmids. Ultimately, a poor correlation exists between the methods when testing unpurified plasmids.

**Table 8.** A_260_ vs. digital droplet PCR (ddPCR) plasmid titer in unpurified plasmids. Quantification of unpurified plasmid by A_260_ and ddPCR plasmid titer. % A_260_ was calculated as the measured ddPCR concentration divided by the A_260_ concentration and expressed as a percentage.

Sample	ddPCR (ng/μL)	A_260_ (ng/μL)	% A_260_
1 (less pure)	21.2	379	6%
2	18.9	301	6%
3	18.9	317	6%
4	18.0	287	6%
5	4.3	63	7%
6	21.9	206	11%
7 (more pure)	212.9	301	69%

## General notes and troubleshooting

**General notes**

Herein, we developed a simplified, one-step ddPCR protocol to evaluate plasmid titer that overcomes the challenges of tertiary structure found in supercoiled plasmid by combining linearization with PCR [3]. Importantly, plasmid conformation has a direct impact on ddPCR performance, as the supercoiled structure can impair primer and probe annealing [3], leading to poor droplet resolution and inaccurate quantification (Figure 1). Incorporation of a linearization step eliminates these effects, resulting in clear droplet separation and reliable copy number determination. Unlike traditional DNA quantification approaches that measure total nucleic acid, ddPCR provides a specific quantification of plasmid copy number that is free from interference by other nucleic acids. Additional advantages of ddPCR include high sensitivity and reproducibility, making it suitable for quantifying both unpurified and purified plasmid across a wide range of concentrations.

The ability of the ddPCR method to accurately quantify plasmid DNA in the presence of nucleic acid contaminants makes it well-suited to support the process of testing plasmid intermediates. However, despite its lower accuracy, the A_260_ method offers advantages in speed and minimal hands-on time. A hybrid approach may therefore be beneficial, using A_260_ for rapid estimation and ddPCR for accurate quantification when needed. A correction factor derived from one sample set was shown to accurately and precisely predict ddPCR concentrations for a second set of similar purity (Table 7), though such factors are matrix-dependent and require validation under consistent conditions.

Overall, the ddPCR method demonstrated high accuracy (104%) and precision (4%), making it well-suited for applications where precise plasmid quantification is critical, such as monitoring plasmid production or determining plasmid input ratios for rAAV TT. More broadly, the method can also be applied as a quality control tool across the plasmid production workflow and adapted for general use through target libraries or matrix-specific correction factors.

**Troubleshooting**

**Problem 1:** Significant droplet rain.

Possible cause: Incomplete linearization of the plasmid template.

Solution: Ensure that the restriction enzyme has cut sites on the target plasmid and confirm by running a Southern blot. Upon confirmation, it may be advisable to either increase the volume of restriction enzyme per ddPCR reaction or increase the duration of the linearization step of the PCR protocol.

**Problem 2:** Target amplicon fails to produce positive droplets.

Possible cause: Beyond typical reasons for ddPCR to fail, the restriction enzyme may have cut inside the target amplicon.

Solution: Evaluate restriction enzyme cut sites in your plasmid of interest using sequence analysis software such as SnapGene. Use another enzyme that produces cuts outside the amplicon if needed.

**Problem 3:** All negative or positive droplets.

Possible cause: Improper sample dilution may have caused copy numbers to fall outside the analytical range.

Solution: Retest samples using either a smaller dilution factor for all negative droplets or a larger dilution factor for all positive droplets.
